# The process of residual calcification following antiparasitic treatment in the pig model of neurocysticercosis is dynamic

**DOI:** 10.1371/journal.pntd.0013022

**Published:** 2025-05-05

**Authors:** Gianfranco Arroyo, Laura Baquedano, Rosa Diaz–Gongora, Lizziee Tello–Ccente, Erick Castillo, Edson Bernal, Omar Gonzales–Viera, Robert H. Gilman, Manuela R. Verastegui, Theodore E. Nash, Armando E. Gonzalez, Hector H. Garcia, Javier A. Bustos, on behalf of The Cysticercosis Working Group in Peru [CWGP]

**Affiliations:** 1 Center for Global Health, Universidad Peruana Cayetano Heredia, Lima, Peru; 2 Carrera de Medicina Veterinaria y Zootecnia, Universidad Cientifica del Sur, Lima, Peru,; 3 Infectious Diseases Laboratory Research–LID, Faculty of Sciences and Philosophy, Universidad Peruana Cayetano Heredia, Lima, Peru; 4 Department of Pathology, Microbiology, and Immunology, School of Veterinary Medicine, University of California, Davis, California, United States of America; 5 Department of International Health, Bloomberg School of Public Health, Johns Hopkins University, Baltimore, Maryland, United States of America; 6 Laboratory of Parasitic Diseases, National Institute of Allergy and Infectious Diseases, National Institutes of Health, Bethesda, Maryland, United States of America; 7 School of Veterinary Medicine, Universidad Nacional Mayor de San Marcos, Lima, Peru; Stony Brook University, UNITED STATES OF AMERICA

## Abstract

**Background:**

Calcified neurocysticercosis (NCC), the end stage of brain cysts of the pork tapeworm *Taenia solium* is a common cause of epilepsy. Calcified NCC lesions are not inert and represent potential epileptogenic foci. Understanding the mechanisms of residual calcification in NCC is hindered by the difficulty of accessing human brain biopsies. Since cyst degeneration can be induced by antiparasitic treatment (APT) in NCC–infected pigs, this study assessed the residual calcification process in this model at three time points after APT.

**Methods/Principal findings:**

Fifteen naturally infected pigs with viable NCC confirmed by magnetic resonance imaging received APT with albendazole and praziquantel and were sacrificed after 4, 8, and 12 months (*n* = 5 each). The pigs’ brains were removed and processed by ex vivo CT scan to assess the proportion of cysts that calcified by post–treatment time points using risk ratios (RR) from Poisson regression. Radiodensity levels (Hounsefield units) of calcified lesions were also measured and compared using linear coefficients from log–transformed values in generalized linear models. The overall proportion of residual calcification on CT scan was 63.9% (156 calcified lesions/244 viable cysts), being statistically higher in treated NCC pigs at 4 months (83.3% [50/60], RR = 2.61, *P *< 0.001) and 8 months (82.8% [77/93], RR = 2.59, *P* < 0.001) versus 12 months (31.9% [29/91]). At 8 months after APT, calcifications were more dense (100.6 ± 3.6 HU) compared to 12 months (74.4 ± 3.6 HU, β *= *0.37, *P* = 0.010) and marginally higher compared to 4 months (85.2 ± 3.8 HU, β = 0.24, *P* = 0.096), and were also larger and more frequently found on histopathology.

**Conclusion/Significance:**

Calcification in NCC is a dynamic process that can be induced and monitored in naturally infected pigs. Eight months after treatment seems to be an optimal time point for assessing residual calcification.

## Summary

Calcified lesions are the most common image finding of neurocysticercosis (NCC) in endemic areas, are considered potential epileptogenic foci and contribute to the pathophysiology of seizure activity in human NCC. A better understanding on the pathophysiological mechanisms of residual calcification in NCC and its association with seizure development are essential. Our findings suggest that the calcification procces is dynamic and can be properly induced by antiparasitic treatment (APT) in naturally infected NCC pigs, as demonstrated by ex vivo computed tomography (CT) scan, histopathology, and scanning–electron microscopy. We also suggest that eight months after APT is an optimal time point for assessing residual calcification. These results provide a reliable animal model for the study of calcified NCC and for assessing controlled interventions in pre–clinical studies aimed to reduce the occurrence of residual calcification.

## Background

The pork tapeworm *Taenia solium* is a zoonotic cestode with a complex lifecycle including humans and pigs [1,2]. Humans serve as definitive hosts of the adult intestinal phase (taeniasis), whereas pigs and humans serve as intermediate hosts of the tissue larval phase (cysticercosis) [1,3]. Neurocysticercosis (NCC) is the infection of the central nervous system (CNS) with *T. solium* cystic larvae and is regarded as a major cause of human acquired epilepsy worldwide [2,4,5]. NCC is a significant public health concern, particularly in developing countries where inadequate sanitary conditions and informal pig farming activities coexist [6,7]. Approximately 30% of epilepsy cases diagnosed in areas endemic for *T. solium* are due to NCC [5,8,9]. NCC is also becoming a more frequent diagnosis in industrialized countries, primarily due to the influx of immigrants and travelers from endemic countries [10–12].

Calcified lesions are the most common finding on brain computed tomography (CT) scan among individuals living in *T. solium*–endemic areas (between 10% to 20%) [13,14]. These lesions appear as small, rounded, hyperdense nodules on CT scan [1,15]. Calcifications are considered the end stage of viable CNS cysts, mainly those located in the brain parenchyma [16]. Once larvae establish as viable cysts in the CNS, they usually cause little or no inflammation in the perilesional tissue probably as a result of parasite–driven modulation of the host’s immune response [1,17,18]. Eventually, either by natural evolution or as a result of antiparasitic treatment (APT) [19], the host’s immune system recognizes the cysts and mounts a robust inflammatory response resulting in cyst degeneration, resolution, or ultimately in the development of residual calcification [19–21]. The rate of spontaneous calcification at 12 months in patients with solitary NCC granulomas ranges between 15% to 56% [22,23], whereas in patients with multiple parenchymal NCC the 12–month rate of residual calcification after APT is about 38% [24]. The exact reason why a dying cysticercus results in a calcified lesion is not known, although some risk factors such as increased cyst size, the presence of perilesional edema, and receiving low doses of corticosteroids during APT have been suggested [22,24].

Calcified lesions in NCC are not quiescent as they were traditionally considered and can act as epileptogenic foci [4,25]. Hospital–based studies reveal seizures in up to 80% of patients with calcified NCC, and in about 50% of these patients seizures are recurrent. Furthermore, population–based studies conducted in NCC endemic areas show statistically significant associations between calcified NCC and epilepsy [26,27], consistently reported in systematic reviews and meta–analysis [4,5,25].

The precise mechanisms for seizure development in calcified NCC remain incompletely known, although it seems to be multifactorial [16], either as a result of blood–brain barrier (BBB) disruption and transient perilesional edema [20,21,28], likely triggered by the sporadic release of parasitic antigen remnants trapped in calcified lesions inducing a strong inflammatory response [29–31], or related to established perilesional tissue alterations and damage such as abnormal vascular permeability, axonal injury, and perilesional gliosis [31]. The association between calcified NCC and hippocampal sclerosis as a contributor for temporal lobe epilepsy has also been reported [32–34]. However, human brain specimens of calcified granulomas for histopathological assessment are not accessible to study these mechanisms. In this context, animal NCC models are needed to study the evolution of the disease, from CNS cyst infection to cyst degeneration and residual calcification, either naturally or induced by APT [35].

The pig is considered the most appropriate animal model for NCC [36]. The pig is the natural host of *T. solium* larvae, and the anatomical and histopathological characteristics of CNS cyst infection in pigs are quite similar to those observed in human NCC [36–38]. Early post–treatment inflammatory responses have been extensively studied in the natural pig model of NCC [39–41]. Pericystic inflammation in the CNS of NCC pigs occurs as early as 48 hours after APT with albendazole (ABZ) and praziquantel (PZQ), being more evident after 120 hours, and has been characterized using brain magnetic resonance imaging (MRI), Evans Blue (EB) staining (EB extravasation), histopathology, and real–time PCR assay (increased expression levels of pro–inflammatory cytokines) [40–44]. However, the longer–term effects of brain inflammation in the NCC pig model and the development of residual calcification using CT scan and histopathology have not yet been investigated. Since cyst death and subsequent degeneration can be induced by APT in the natural pig model of NCC, this study aimed to evaluate the development of residual brain calcification in this model at three different time points after APT.

## Materials and methods

### Ethics statement

The study protocol was previously reviewed and approved by the Institutional Committee for Animal Use and Care at the Universidad Peruana Cayetano Heredia (approval number R-036-12-18). All the study procedures were conducted in accordance with the standard guidelines of the Association for Assessment and Accreditation of Laboratory Animal Care (AAALAC/NIH).

### Study design and objectives

This unblinded, pre–clinical study assessed the development and characteristics of residual brain calcifications in the natural pig model of NCC at 4, 8, and 12 months after APT using ex vivo CT scan, histopathology, and scanning–electron microscopy (SEM) in order to confirm the usefulness of the naturally infected pig model for this purpose, and also to select the most appropriate post–treatment time point to detect calcifications, as a key parameter to design further studies to evaluate factors affecting residual calcification.

### Animals and facilities

Twenty-one rural pigs with cysticercosis determined by tongue examination [45] and confirmed by positive result on serum enzyme–linked immunoelectrotransfer blot (EITB) assay for anti–*T. solium* antibody detection [45,46] were purchased from rural endemic communities of Tumbes and Huancayo, Peru [6,47]. Pigs were between 9–12 months of age, either male or female, and weighted between 40–90 kg. Animals were transported to our pathogen–free veterinary facilities at the School of Veterinary, Universidad Nacional de San Marcos in Lima, Peru. Pigs were kept for a 2–week acclimatization period in appropriate conditions, with cycles of 12/12 light and dark hours, were vaccinated against hog cholera, and received food and water *ad libitum.*

### Study activities

#### Brain magnetic resonance imaging (MRI).

**Fig 1:**
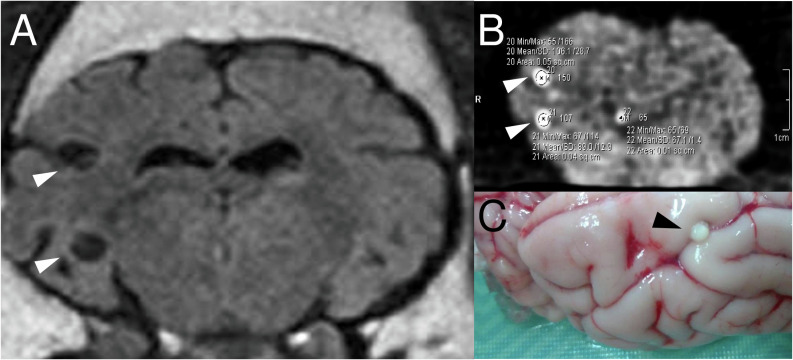
Basal brain MRI exam showing viable cysts (A); ex vivo CT scan showing calcified lesions with measurements of radiodensity (HU) and areas (cm^2^) (B); apparent calcified lesion identified on gross exam (C).

All pigs underwent a basal MRI exam to verify the presence of viable brain cysts. The MRI exam was performed at the Instituto Nacional de Ciencias Neurologicas (INCN) in Lima, Peru, using a 3–Tesla scanner (Phillips, Achieva, Best, Netherlands). The MRI protocol included axial and coronal FLAIR (fluid–attenuation inverse recovery) and T2–weighted sequences at 3 mm of slice thickness with no gap. Before MRI exam, all pigs were induced to anesthesia using a combined intramuscular dose of ketamine (10 mg/kg, Ket–A 100, Agrovet Market, Peru) plus xylazine (2 mg/kg, Dormi–Xyl, Agrovet Market, Peru), and maintained by continuous intravenous infusion of ketamine (5 mg/kg). Viable brain cysts appeared on MRI as small, rounded, low–intensity areas well demarcated from the surrounding brain tissue, and showing a hyperintense nodule in its interior representing the scolex (Fig 1A) [44,48]. The number and topographic location of viable brain cysts in each pig were recorded.

#### Antiparasitic treatment and follow-up.

All pigs with NCC determined on basal MRI received a combined APT scheme using ABZ (Sanibendazole 10%, Montana, Peru, 15 mg/kg divided in two doses per day, for 5 days) plus PZQ (Saniquantel 10%, Montana, Peru, 75 mg/kg divided in three doses per day for 1 day). The dose of antiparasitic drugs administered per pig was calculated according to pigs’ weight measured in an automated scale. Antiparasitic drugs were administered by veterinary technicians using an oral canula, and under the supervision of the study veterinarian. Immediately after APT, all pigs were monitored for approximately 2 hours to register possible regurgitation or any adverse effect such as asphyxia due to aspiration of the drugs. From then on, the clinical status of the pigs was registered every day by the veterinary staff throughout the study period.

#### Allocation of NCC pigs in experimental groups.

Two weeks after APT, NCC pigs were allocated into three homogeneous groups according to CNS parasitic load determined on basal MRI). The assigned groups corresponded to three post–treatment time points for necropsy (4, 8, and 12 months, respectively).

#### Necropsy, brain perfusion and extraction.

Pigs were sacrificed according to the post–treatment time points specified for each experimental group. For euthanasia, pigs were sedated using a combined intramuscular dose of ketamine plus xylazine (as previously described for MRI exam) and subsequently received an intravenous overdose of sodium pentobarbital (Penta–Hypnol, 120 mg/kg, Agrovet Market, Peru). Immediately after euthanasia, the pig brain was perfused with 3000 mL of a chilled saline solution (0.85% NaCl) with heparin (10 UI/mL) via catheterization of the right common carotid artery connected to a peristaltic pump. The pig brain was removed from the skull and placed in a plastic container with 1% Phosphate–Buffered Saline (PBS) solution.

#### Ex vivo brain computed tomography (CT) scan.

The pig brain was processed by ex vivo CT scan at the INCN, Lima, Peru using a Somatom Sensation 64 Eco–used CT scanner (Siemens Healthineers, Germany) to obtain contiguous coronal cranial sections of 3 mm of slice thickness of the whole brain. Findings from CT scan included the number and topographic location of calcified lesions (visualized as hyperdense small, well demarcated dots or punctate lesions, with or without perilesional edema, Fig 1B). Hounsfield units (HU) were also calculated for each calcified lesion as a relative quantitative measurement of radiodensity using the absortion/attenuation linear radiation coefficient of the X–ray beam to produce a grayscale image. For this calculation, a region of interest (ROI) was manually delimited in each calcified lesion visible on CT scan and average HU values of radiodensity and lesions areas (mm^2^) were automatically obtained in the Syngo.via software (Siemens Healthineers, Forccheim, Germany).

#### Brain tissue collection.

The entire brain was cut into 5 mm coronal sections. We collected brain biopsies in areas where we identified lesions consistent with calcifications (small, well–defined, whitish, rounded lesions, Fig 1C), taking as a reference the findings of ex vivo CT scan. Biopsies were coded and fixed separately in 10% neutral–buffered formalin (3.7% formaldehyde in PBS, pH = 7.2) for 24 hours, dehydrated in an alcohol series, and paraffin embedded. Brain sections were cut consecutively into 4 µm thick sections until reaching the central section of each lesion (that with the largest diameter). Central sections were mounted in Poly–L–lysine coated slides for histopathological assessment.

#### Histopathology and confirmation of calcified granulomas.

Conventional Hematoxylin–Eosin (H&E) and Masson’s Trichome (MTC) stain methods were performed on paraffin embedded sections to determine the presence of granulomas (lesions characterized by the presence of parasite remnants, necrotic tissue in its interior and surrounded by inflammatory cellular infiltrates consisting of lymphocytes, clusters of macrophages, epithelioid and/or multinucleated cells and fibrotic layers) or fibrotic lesions (significant accumulation of collagen fibers, absence or low number of inflammatory cells, and alterations in the surrounding blood vessels such as hyalinization or thickening of vessel wals). Subsequently, granulomas were processed using Alizarin–Red (AR) stain to evaluate the presence of calcium deposits and calcified matrix inside lesions using the methodology described by Bancroft & Gamble [49]. Calcified granulomas were classified according to their location in the brain tissue as parenchymal, corticomeningeal or extraparenchymal.

#### Scanning–electron microscopy (SEM).

A subsample of calcified granulomas were randomly selected, fixed in 10% glutaraldehyde, dehydrated with ethanol, cut into 3 µm sections and placed on a copper grid for SEM assesssment. Images were captured using a FEI–Quanta 200 FEG SEM microscope (Hillsboro, OR, USA). The total area of the calcified granuloma was evaluated to quantify the composition of chemical elements (calcium [Ca], phosphorus [P], carbon [C], oxygen [O], sodium [Na], and magnesium [Mg]) using energy–dispersive X–ray spectroscopy (EDX) and expressed as weight percentages (Wt%).

### Study variables

The main outcome variable was measured at the pig level and consisted of the proportion of brain cysts that resulted in calcified lesions determined by ex vivo CT scan (hyperdense lesions with >50 HU values). Secondary outcomes from ex vivo CT scan in calcified lesions were the measurement of radiodensity (HU values) and lesion area (mm^2^, both included as continuous variables). We also reported the proportion of calcified granulomas by AR–stain among processed brain biopsies. Outcomes from SEM included the weight percentages of chemical elements per sample.

### Statistical analysis

Study variables were described using summary statistics (frequencies and percentages for categorical variables and mean ± standard errors [SE] or median and interquartile ranges [IQR] for numerical variables) and distributed according to groups (time points after APT). We compared the proportion of residual calcification in treated NCC pigs by post–treatment time points using risk ratios (RR) with 95% confidence intervals obtained from Poisson regression models. Mean HU values and areas (log–transformed values) of calcified lesions measured on ex vivo CT scan were also compared between post–treatment time points using linear regression coefficients obtained from generalized linear models (GLM) with gaussian family distribution, and identity link function, whereas the proportion of calcified granulomas on AR–stain over the total number of brain biopses processed was also compared by groups using RRs from log–binomial GLMs. Regression models used clustered robust–variance estimates to account for the correlation of sample units per pig brain. SEM findings (Wt% of chemical elements) were compared between groups using non–parametric tests clustered by pig brain. All the statistical analyses were carried out in RStudio v1.4.1106, using a significance level of 5% for comparisons. Plots were created using the software GraphPad Prism version 9.5.1 (GraphPad software, LLC).

## Results

Twenty–one pigs naturally infected with cysticercosis determined by tongue exam and EITB were purchased for this study, of which, 13 (61.9%) were female. On baseline MRI, 18/21 pigs (85.7%) had viable NCC and were included in the study. These pigs had a median of 15 viable brain cysts (IQR: 6–29); and had a median of 7 reactive antibody bands on EITB (IQR: 6–7).

All pigs with NCC received combined APT with ABZ plus PZQ. The clinical status of the pigs was monitored during APT. Three pigs died throughout the study period; the two pigs with the largest CNS cyst burden (60 and 100 brain cysts) were found dead on days 3 and 4 of APT, whereas a third pig was euthanized two weeks after APT onset due to an episode of pneumonia that did not improve despite medication. The remaining 15 NCC pigs did not show evident neurological signs such as seizures, abnormal behaviour (e.g., salivation, twitching, trembling, etcetera) or discomfort during APT. These pigs were re–distributed to keep three experimental groups of 5 pigs each. A total of 244 viable brain cysts were found on MRI in these 15 pigs (60, 93, and 91 cysts at 4, 8, and 12 months, respectively). Viable cyst burden per pig was not statistically different between groups (median: 11 cysts [IQR: 6–14], median: 15 cysts [IQR: 6–18], and median: 17 cysts [IQR: 8–29] at 4, 8, and 12 months, respectively, *P* = 0.635, Table 1).

**Table 1:** Findings from basal MRI (viable brain cysts) and ex vivo CT scan (calcified lesions), and risk ratios for the proportion of residual calcification in NCC pigs according to time points after APT (*n* = 5 pigs in each group).

Time point after APT	Nº of viable cysts on basal MRI			Nº of calcified lesions on ex vivo CT scan			Proportion of residual calcification		
	Total	Median (IQR)	*P*	Total	Median (IQR)	*P*	C/Cy (%)	RR* (95% CI)	*P*
4 months	60	11 (6–14)	0.635	50	6 (2–16)	0.648	50/60 (83.3)	2.61 (1.65 to 4.13)	0.001
8 months	93	15 (6–18)		77	2 (2–21)		77/93 (82.8)	2.59 (1.69 to 3.98)	0.001
12 months	91	17 (8–29)		29	2 (1–2)		34/91 (31.9)	Ref.	–
Total	244	14 (6-26)	–	165	2(1–21)	–	161/244 (65.9)	–	–

Abbreviations: APT (antiparasitic treatment), MRI (magnetic resonance imaging), CT (computed tomography); IQR (interquartile range); C (calcified lesios); Cy (viable cysts), RR (risk ratios)

* RRs for residual calcification were obtained from Poisson regression models.

Ex vivo CT scan demonstrated the presence of at least one calcified lesion in 13/15 pigs (86.7%) after APT. The overall proportion of residual calcification after APT was 63.9% (156 calcified lesions on CT scan/ 244 viable cysts on basal MRI). We observed higher proportions of residual calcification in treated NCC pigs at 4 and 8 months (83.3% [50/60] and 82.8% [77/93]) compared with treated NCC pigs at 12 months (31.9% [29/91], RR = 2.61 [95% CI: 1.65 to 4.13], *P *< 0.001 for 4 months and RR = 2.59 [95% CI: 1.69 to 3.98], *P* < 0.001 for 8 months, Table 1).

Quantitative results from ex vivo CT scan are shown in Table 2. Calcified lesions had a mean area of 4.6 ± 0.4 mm^2^ (ranging from 1 to 32 mm^2^). Mean calcified lesion areas from treated NCC pigs at 8 months were larger albeit more variable in size (mean ± SE: 6.4 ± 0.8 mm^2^) than those at 12 months (mean ± SE: 4.40 ± 0.4 mm^2^) or those at 4 months after APT (mean ± SE: 2.2 ± 0.2 mm^2^). The difference in the mean area of calcifications between the 4– and 12–month groups was statistically significant in the regression analysis (β = 0.69 [95% CI: 0.49 to 0.89], *P* < 0.001). Calcified lesions from treated NCC pigs were more dense at 8 months (mean radiodensity: 100.6 ± 3.6 HU) compared to 4 months (mean radiodensity: 85.2 ± 3.8 HU) and 12 months (mean radiodensity: 74.4 ± 3.6 HU). The regression analysis using log–transformed HU values showed statistically higher radiodensity in calcified lesions at 8 months compared to 12 months (β *= *0.37 [95% CI: 0.09 to 0.66], *P* = 0.010) and marginally higher radiodensity levels in calcified lesions at 4 months compared to 12 months (β = 0.24 [95% CI: -0.04 to 0.52], *P* = 0.096).

**Table 2:** Radiodensity levels (Hounsfield units) and areas (mm^2^) of calcified lesions measured on ex vivo CT scan in treated NCC pigs at 4, 8, and 12 months.

Time point after APT	Nº of calcified lesions on CT scan	Lesion area (mm^2^)			Radiodensity (HU)		
		Mean ± SE	β* (95% CI)	*P*	Mean ± SE	β* (95% CI)	*P*
4 months	50	2.2 ± 0.2	-0.69 (-0.89 to -0.50)	<0.001	85.2 ± 3.8	0.12 (-0.10 to 0.34)	0.096
8 months	77	6.4 ± 0.8	0.02 (-1.03 to 1.06)	0.975	100.6 ± 3.6	0.29 (0.02 to 0.57)	0.048
12 months	29	4.4 ± 0.5	Ref.	–	74.4 ± 3.6	Ref.	–
TOTAL	156	4.6 ± 0.4			90.8 ± 2.4		

Abbreviations: HU (Hounsfield unit); APT (antiparasitic treatment); CT (computed tomography); CI (confidence interval); SE (standard error).

* β coefficients with 95% CI were obtained from generalized linear models (GLMs) using Gaussian family distribution, identity link-function and clustered robust-variance estimates to account for the correlation of calcified lesions per pig brain.

A total of 140 brain biopsies were collected, embedded in paraffin and processed by histopathology using H&E and MTC stain. Among these, 63 (45.0%) showed defined characteristics of granulomas, whereas the remaining biopsies (77, 55.0%) were classified as fibrotic lesions on H&E and MTC stains. AR stain was performed under similar coditions on lesions identified as granulomas and confirmed the presence of calcium–rich deposits in 43/63 samples (68.3%); therefore, the overall proportion of calcified granulomas among all brain biopsies was 30.7% (43/140), being statistically higher in treated NCC pigs at 8 months (26/64 [40.6%]) versus 12 months (17.0% [8/47], RR = 2.39, [95% CI: 1.05 to 5.42], *P* = 0.038) but not versus 4 months (9/29 [31.0%], RR = 1.30 [95% CI: 0.44 to 3.92], *P* = 0.630, Table 3). Most calcified granulomas had parenchymal and corticomeningeal location (21/43 [48.8%] and 18/43 [41.9%]) whereas only 4 calcified granulomas had extraparenchymal location.

**Table 3:** Proportion of calcified granulomas confirmed by Alizarin–Red stain among brain biopsies collected from treated NCC pigs at 4, 8, and 12 months.

Time point after APT	Nº of brain biopsies collected	Nº of calcified granulomas confirmed by AR stain	Proportion of calcified granulomas		
C_G_/B_B_ (%)	*RR (95% CI)	*P*
4 months	29	9	31.0	1.82 (0.68 to 4.87)	0.231
8 months	64	26	40.6	2.39 (1.05 to 5.43)	0.038
12 months	47	8	17.0	Ref.	–
TOTAL	140	43	30.7	–	–

Abreviations: AR (Alizarin-Red); RR (risk ratio); C_G_ (calcified granulomas); B_B_ (brain biopies); CI (confidence interval)

*RRs with 95% CI were obtained from log-binomial Generalized Linear Models (GLM) with clustered robust-variance estimates to account for the correlation of brain biopsies per pig brain.

**Fig 2:**
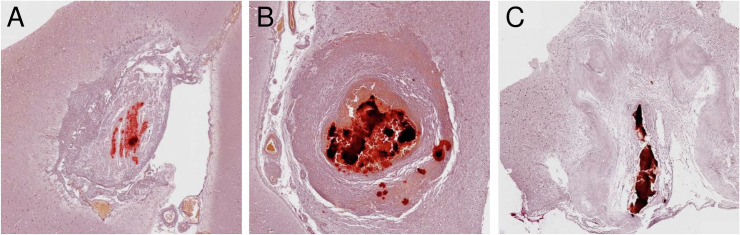
Microscopic visualization of calcium aggregates by Alizarin–Red (AR) stain in calcified granulomas from treated NCC pigs at 4, 8. and 12 months (A, B, and C).

Microscopic evaluation of AR–processed calcified granulomas from treated NCC pigs at 4 months showed a characteristic pattern of diffuse and amorphous mineralization (Fig 2A), which was more evident and diffuse inside the calcified granulomas at 8 months after treatment (Fig 2B). Calcium aggregates decreased in calcified granulomas from treated NCC pigs at 12 months and were replaced by tortuous bands of mature connective tissue (Fig 2C). A lower proportion of calcified granulomas showed a concentric pattern of rounded–small calcifications representing the calcareous corpuscles.

**Fig 3:**
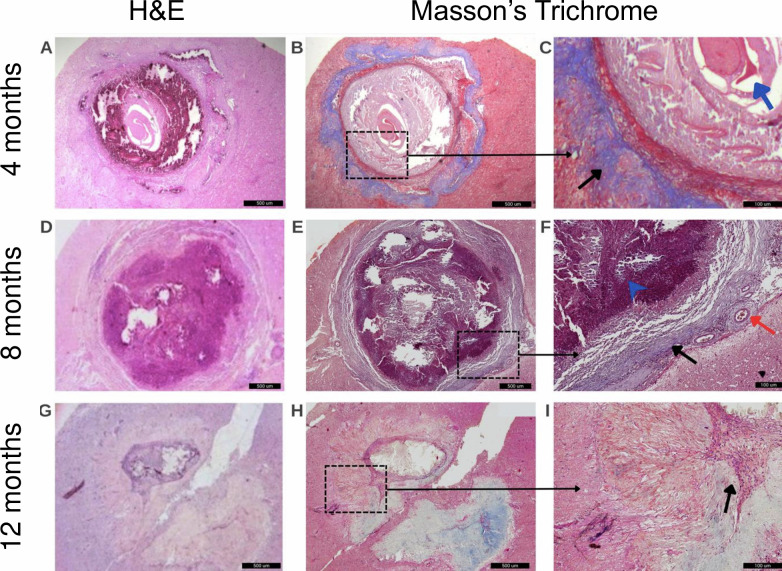
Inflammatory and fibrotic responses in calcified granulomas from treated NCC pigs at 4 (A–C), 8 (D–F), and 12 (G-I) months. H&E (A, D, G) and Masson’s Trichrome (B, E, H) stains. (C) Silhouette of a cysticercus with a central scolex is observed and partially effaced by dystrophic mineralization (blue arrow). Surrounding the degenerate metacestode is a semi dense and tortuous band of mature fibrous connective tissue (black arrow). (F) The cysticercus is completely effaced by large amounts of dystrophic mineralization and cellular necrotic debris surrounded by numerous lymphocytes (arrowhead), plasma cell and some histiocytes embedded in in a disperse and immature fibrous connective tissue (black arrow). The adjacent blood vessels are reactive and have lymphocytic perivascular cuffing (red arrow). (I) The metacestode is surrounded by some lymphocytes and plasma cells embedded in an immature fibrous connective tissue and externally sequestered by a dense, thick, and disorganized mature fibrous connective tissue (arrow). (A, B, D, E, G, H, 5X magnification) (C, F, I, 20X magnification).

Histopathological evaluation of calcified granulomas revealed different degrees of inflammatory and fibrotic responses in the perilesional tissue according to post–treatment time points in H&E and MTC stains (Fig 3A–I). At 4 months after APT, most calcified granulomas showed signs of effacement due to dystrophic mineralization, with only fragments of scolex hooks or a cyst silhouette with a central scolex surrounded by multinucleated cells, lymphocytes, plasma cells, and tortuous band of at immature fibrous connective tissue (Fig 3A–C). At 8 months after APT, calcified granulomas were partially or completely effaced by dystrophic mineralization, with presence of necrosis and surrounded by a variety of inflammatory cells including lymphocytes, plasma cells, histiocytes, and multinucleated giant cells; signs of neovascularization in the surrounding tissue as well as dense bands of mature fibrous connective tissue with some areas showing whorls and projections into the neutrophil were also observed (Fig 3D–F). By 12 months after APT, calcified granulomas showed advanced degeneration characterized by extensive mineralization and effacement and were surrounded by varying degrees of inflammatory cell infiltrates including lymphocytes, plasma cells, multinucleated giant cells, whereas the external layer was sequestered by a dense, thick, and disorganized mature fibrous connective tissue (Fig 3G–I).

**Fig 4:**
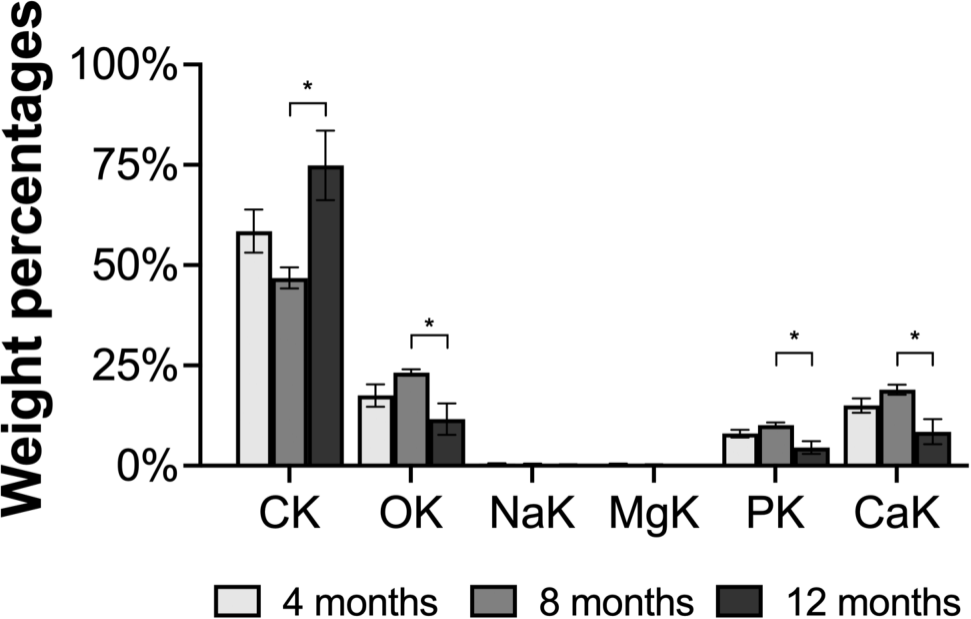
Bar plots showing the mean ± standard error of element composition of calcified granulomas (expressed as weight percentages [Wt%]) by scanning–electron microscopy (SEM) in treated NCC pigs at 4, 8, and 12 months. Abbreviations: CK (carbon); OK (oxygen); NaK (sodium); MgK (magnesium); PK (phosphorus); CaK (calcium). Bars represent means and whiskers represent standard errors. * indicates *P* < 0.05 for comparisons by post-treatment time points.

Scanning–electron microscopy was performed in 18 randomly selected calcified granulomas (*n *= 5, 9, and 4 lesions from treated NCC pigs at 4, 8, and 12 months, respectively, Fig 4). Calcium concentrations (Wt%) were statistically higher at 8 months after APT (mean ± SE: 19.0 ± 1.2) versus 12 months (mean ± SE: 8.5 ± 3.1; *P* = 0.006); and also, higher (but not statistically significant) when compared with the 4 months group (mean ± SE: 15.0 ± 1.8, *P* = 0.102). The concentrations of phosphorus were also higher at 8 months and 4 months, but lower at 12 months, and statistically different (*P* = 0.032), and a similar trend was found for the concentrations of oxygen (*P* = 0.037). On the other hand, carbon concentrations (Wt%) were statistically higher at 12 months (mean ± SE: 74.9 ± 8.7) compared to 8 months (mean ± SE: 46.8 ± 2.7 *P* = 0.006), but not different when compared with 4 months (mean ± SE: 58.5 ± 5.4; *P *= 0.112). Sodium and magnesium concentrations represented the lowest chemical composition in calcified granulomas (<1% of Wt%), and their concentrations were not different by post–treatment time point.

## Discussion

Calcified NCC has a significant impact on public health due to its chronic nature, with seizures and headaches being the most frequent symptoms that can persist for years in the patient [17,50,51], seriously affecting the patient’s quality of life [52]. Our findings confirm that calcificied cerebral cysts are not quiescent, inactive lesions and demonstrate the dynamic nature of the development of residual brain calcification in NCC. Our data also suggests that 8 months after APT is an appropriate time point to assess the long-term effects and consequences of residual calcification in the CNS and subsequently testing therapeutic strategies for its control.

The mechanisms behind the development of residual calcification in NCC remain poorly known. Most studies of residual calcification in patients with NCC are based on CT scan; these studies reported between 20% and 56% of spontaneous residual calcification in patients with solitary NCC granulomas [22], whereas in patients with multiple viable NCC the rate of calcification 12 months after APT is around 38% [24]. In our study, the overall proportion of residual calcification seen on *ex vivo* CT scan was 63.9%, being higher at 4 and 8 months after APT (83.3% and 82.8%), and lower at 12 months (31.9%). However, there are differences in the conditions leading to residual calcification in treated NCC pigs in our study versus those that occur in human NCC. For example, CNS cysts in human NCC are very likely older and bigger compared with those in porcine NCC (usually pigs in rural areas live 9–12 months) and may harbor some degree of pre-existing inflammation. Also, pigs developed calcified NCC after APT without the concomitant use of steroid anti-inflammatory drugs. Corticosteroids are frequently administered during APT in patients with viable NCC to reduce the risk of seizures due to acute brain inflammation, and a previous study by our group demonstrated that increasing the dose of corticosteroids during APT in viable NCC reduces the likelihood of residual calcification [16,24]. For this reason we considered to conduct the experiment without the interference of corticosteroids in order to directly evaluate the effects of treatment–induced neuroinflammation on the residual calcification process. It is likely that the two pigs with the highest parasitological loads have died due to a severe neuroinflammatory process after APT, although we cannot ruled out other causes such as side–effects of albendazole of pigs (toxicity, and leukopenia) [53].

Ex vivo CT scan allowed us to identify calcified lesions in treated NCC pigs. Previous studies reported a high sensitivity of CT scan for the diagnosis of calcified NCC (almost 100%). There were more calcified lesions detected on ex vivo CT scan than those identified on gross examination. Considering the exaggerated sensitivity of CT to mark calcium due to some factors such as beam hardening artifacts or partial volume effect [54], it is possible that some calcified lesions visualized on ex vivo CT scan were too small to be detected on gross exam, as observed in treated NCC pigs at 4 months who had high number of calcified lesions on CT scan, but with the smallest mean area (<2 mm^2^) and were the group of NCC pigs with the lowest number of samples collected for histopathology (20.7% of the total brain biopsies). In any case, CT scan provides a practical standard of reference for comparative pre-clinical and clinical studies. Brain biopsies of human NCC lesions are rarely available for histopathological assessment.

Eight months after the onset of APT seems to be the optimal time point to evaluate the residual calcification process in NCC pigs. In this group there was a higher proportion of calcified lesions on CT scan, a higher proportion of calcified granulomas evidenced, and higher concentrations of calcium in the lesions. Amorphous and diffuse calcifications were the predominant type of calcification observed in treated NCC pigs (mainly in treated NCC pigs at 8 months), which contrast with a pattern of calcification characterized by dense concentrations of calcareous corpuscles reported by Nash et al [31] and Klotz et al [55] in selected human biopsies. Amorphous and diffuse calcifications have also been described in the older literature [54], and may represent the predominant pattern of calcified lesion induced by APT in NCC since there are no systematic histopathological studies characterizing the calcification process in humans. On the other hand, most of the calcified lesions from treated NCC pigs at 12 months showed a decrease in the amount of calcium aggregates and granulomatous structures were replaced by connective tissue.

The process of residual calcification in NCC is dynamic, as previously suggested in neuroimaging and histopathological–based human studies [23,31]. The observed differences in the proportion and radiodensity levels of calcified lesions on CT scan, and the characteristics of calcium aggregates and inflammatory responses in calcified granulomas from NCC pigs treated at 4, 8, and 12 months clearly demonstrate temporal patterns of change indicating a slow degeneration process as suggested by Gupta et al [56] and Ooi et al [30]. Evidently not all cysts calcify, and not all cysts that calcify do it at the same time. There were defined parasite remnants in calcified granulomas at 4, 8, and 12 months after APT, similar to those observed on histopathological descriptions of calcified granulomas from human NCC cases [29], suggesting that some brain cysts did not undergo complete degradation, and supporting the idea that sporadic antigens from parasite remnants trapped in calcified lesions may be released and be the cause of perilesional edema in calcified NCC, a factor related to seizure development [16,31].

This study has some drawbacks. First, the differences in the proportion and radiodensity levels of calcified lesions between groups of treated NCC pigs may represent intrinsic inter–pig variability; for this reason, all the comparative analyses accounted for the correlations between tissue samples per pig brain. Second, baseline imaging in NCC pigs only used MRI and not CT because MRI is highly sensitive in detecting viable brain cysts in pigs, especially when using T2-weighed and FLAIR sequences as previously reported [48,57]. Thus, although unlikely, some of the studied lesions could have been already calcified before APT and have been missed on MRI since its performance to detect calcific lesions is lower compared to CT scan. In the same way, we only used ex vivo CT scan to evaluate calcified lesions at post–treatment time points after APT since treatment success (in terms of viable brain cysts) was not the purpose of the study, and because eventual viable cysts could be identified on gross examination. Third, uncertainties about cyst longevity and pre–existing inflammation in naturally infected NCC pigs may also affect the precision of the comparisons between groups, although these factors also occur in human NCC since infection is more chronic. Replicating our findings in experimental porcine NCC models where the timeline between infection and cyst development is clearly defined may allow more precise comparisons. Also, the lack of an untreated control group may represent a limitation in our study design, although the evidence of previous experimental studies indicates that untreated pigs may remain with viable cysts for up to 12 months as demonstrated by the high antigen levels [58,59]. Finally, the differences in the proportions of calcified granulomas confirmed on AR-stain among brain biopsies assessed between groups may be biased due to selection of lesions identified on gross examination.

The mechanisms associated to the development of epilepsy in calcified NCC have not yet been fully studied. Calcified lesions in NCC can be enduring epileptic foci [16,21], and therefore a better understanding of the pathophysiological processes related to the development of residual calcification in NCC is crucial. A suitable model of calcified NCC will allow researchers to obtain data on chronic neuroinflammation, brain damage, and to define the mechanisms underlying neuronal damage such as autophagy, apoptosis, axonal transport, and their interaction in epileptogenesis, quite limited in studies involving human brain specimens.

Our findings suggest that the calcification process is quite dynamic in its nature and can be properly induced by APT in the natural pig model of NCC as observed by *ex vivo* CT scan, histopathology, and SEM, also suggesting 8 months after APT as an optimal time point for assessing residual calcification. Further studies using longer assessment time–points after APT onset (more than 12 months) will also allow to evaluate the evolution of calcified lesions in the porcine NCC model. In summary, the porcine model of NCC is a suitable model for studying the pathophysiological mechanisms of residual calcification, and may provide a more appropriate model system to test novel pharmacological intervention aimed to reduce the the occurrence of residual calcification.

## Supporting information

S1 Data Data files. (XLS)
